# Attribute-centred theorizing to address behavioural changes

**DOI:** 10.2471/BLT.20.285363

**Published:** 2021-08-30

**Authors:** Rajiv N Rimal, Maria Knight Lapinski

**Affiliations:** a Department of Health, Behavior and Society, Johns Hopkins University, 624 N. Broadway, No. 702, Baltimore, MD 21205, United States of America (USA).; b Department of Communication, Michigan State University, East Lansing, USA.

## Abstract

Despite the importance of behaviours in promoting health and wellness, persuading people to adopt and sustain healthy behaviours remains a significant public health challenge. Considerable progress has been made in developing and testing theories about the personal, social, environmental and structural drivers of behaviours. However, theorizing about behaviours themselves has remained elusive, as evidenced by the absence of a widely accepted taxonomy of behaviours. By carefully examining the nature of behaviours, practitioners and researchers can identify the most effective ways to promote behavioural change. We propose attribute-centred theorizing as an approach for defining behaviours based on their relevant properties, which can then assist in developing a taxonomy of behaviours and theorizing about them. Behaviours differ because of their underlying properties; for example, some behaviours are addictive, others are publicly observable and others are expensive. Addictiveness, privacy and cost are therefore three (of the many) attributes relevant for theorizing about behaviours. We describe a framework for operationalizing attribute-centred theorizing, which includes generating behavioural attributes, verifying and testing those attributes, and constructing a behavioural matrix to inform campaigns or interventions. We illustrate this framework using the examples of Guinea-worm disease and cardiovascular diseases. The benefits of our approach include the ability to inform intervention development and the ability to generalize across different behaviours; however, more research on converting the behavioural matrix into actual policy is needed.

## Résumé

Malgré l'importance des comportements dans la promotion de la santé et du bien-être, persuader les gens d'adopter et de conserver un mode de vie sain représente toujours un défi de santé publique non négligeable. Des progrès considérables ont été accomplis en ce qui concerne le développement et l'évaluation des théories sur les motivations personnelles, sociales, environnementales et structurelles exerçant une influence sur les comportements. Cependant, formuler des théories à ce propos demeure complexe, comme en atteste l'absence de taxinomie largement admise en la matière. En examinant attentivement la nature des comportements, les chercheurs et praticiens peuvent identifier les moyens les plus efficaces pour les faire évoluer. Nous proposons d'opter pour une théorisation axée sur les attributs afin de définir les comportements en fonction de leurs propriétés utiles, ce qui permettrait ensuite d'instaurer une taxinomie des comportements et d'énoncer les hypothèses qui en découlent. Les comportements varient en raison de leurs propriétés sous-jacentes; certains par exemple sont liés à une dépendance, d'autres sont clairement visibles et d'autres encore sont onéreux. La dépendance, l'intimité et le coût constituent donc trois (des nombreux) attributs utiles à l'élaboration d'hypothèses sur les comportements. Dans le présent document, nous traçons un cadre servant à concrétiser cette théorisation axée sur les attributs; il prévoit notamment de générer des attributs comportementaux, de vérifier et de tester ces attributs, et d'imaginer une matrice qui fournirait des orientations pour diverses campagnes ou interventions. Nous illustrons également ce cadre en prenant pour exemples la dracunculose et les maladies cardiovasculaires. Parmi les avantages de notre approche figurent la possibilité de guider le développement des interventions et celle d'intégrer différents comportements; néanmoins, une étude approfondie est nécessaire pour définir comment convertir la matrice comportementale en réelle politique.

## Resumen

A pesar de la importancia de los comportamientos en la promoción de la salud y el bienestar, persuadir a las personas para que adopten y mantengan comportamientos saludables sigue siendo un importante reto para la salud pública. Se ha avanzado considerablemente en el desarrollo y la comprobación de teorías sobre los factores personales, sociales, ambientales y estructurales que impulsan los comportamientos. Sin embargo, teorizar sobre los comportamientos en sí sigue siendo difícil, como demuestra la ausencia de una taxonomía de comportamientos ampliamente aceptada. Al examinar cuidadosamente la naturaleza de los comportamientos, los profesionales e investigadores pueden identificar las formas más eficaces de promover el cambio de comportamiento. Proponemos una teoría centrada en los atributos como enfoque para definir los comportamientos en función de sus propiedades relevantes, lo que puede ayudar a desarrollar una taxonomía de comportamientos y a teorizar sobre ellos. Los comportamientos difieren por sus propiedades subyacentes; por ejemplo, algunos comportamientos son adictivos, otros son observables públicamente y otros son costosos. La adicción, la privacidad y el coste son, por tanto, tres (de los muchos) atributos relevantes para teorizar sobre los comportamientos. Describimos un marco para hacer operativa la teoría centrada en los atributos, que incluye la generación de atributos de comportamiento, la verificación y comprobación de dichos atributos y la construcción de una matriz de comportamiento para informar sobre campañas o intervenciones. Ilustramos este marco con los ejemplos de la enfermedad del gusano de Guinea y las enfermedades cardiovasculares. Las ventajas de nuestro enfoque son la capacidad de informar sobre el desarrollo de intervenciones y la capacidad de generalizar entre diferentes comportamientos; sin embargo, es necesario investigar más sobre la conversión de la matriz de comportamiento en una política real.

## ملخص

على الرغم من أهمية السلوكيات في تعزيز الصحة والسلامة، فإن إقناع الناس بتبني السلوكيات الصحية والحفاظ عليها يظل من الصعوبات الكبيرة في مجال الصحة العامة. لقد تحقق تقدم كبير في وضع نظريات واختبارها بشأن الدوافع الشخصية والاجتماعية والبيئية والهيكلية للسلوكيات. إلا أن وضع النظريات التي تتناول السلوكيات نفسها ظل خادعًا كما يظهر في غياب تصنيف مقبول على نطاق واسع للسلوكيات. عند فحص طبيعة السلوكيات بعناية، يستطيع الممارسون والباحثون تحديد الطرق الأكثر فعالية لتعزيز التغير السلوكي. نطرح النظريات المرتكزة على السمات باعتبارها نهجًا لتعريف السلوكيات بناءً على خصائصها ذات الصلة، وهو الأمر الذي سيساعد بدوره على وضع تصنيف للسلوكيات والنظريات المتعلقة بها. تختلف السلوكيات بسبب خصائصها الكامنة، فبعض السلوكيات مثلاً إدمانية، وهناك سلوكيات أخرى ظاهرة علنًا وأخرى مكلفة. الإدمان والخصوصية والتكلفة إذن ثلاث من السمات (الكثيرة) ذات الصلة بوضع النظريات المتعلقة بالسلوكيات. نصف إطار عمل لتفعيل وضع النظريات المرتكزة على السمات، والذي يتضمن إنشاء سمات سلوكية، والتحقق من تلك السمات واختبارها، وإنشاء مصفوفة سلوكية لتوعية الحملات أو التدخلات. نوضح إطار العمل هذا باستخدام داء الدودة الغينية وأمراض القلب والأوعية الدموية كمثالين. تشمل الفوائد التي ينطوي عليها نهجنا القدرة على التوعية لوضع تدخل والقدرة على التعميم على السلوكيات المختلفة، إلا أن هناك حاجة لإجراء المزيد من الأبحاث على تحويل مصفوفة السلوكيات إلى سياسة فعلية.

## 摘要

尽管行为对改善身心健康起到重要的作用，但说服人们采取和维持这些健康的行为仍然是一个巨大的公共卫生挑战。我们在开发和测试行为的个人、社会、环境和结构性驱动因素理论方面已经取得了重大进展。然而，仍然难以构建关于行为本身的理论，缺乏广泛接受的行为分类法就证明了这一点。通过细心研究行为的性质，从业者和研究者可以确认促进行为改变的最有效方法。我们提出以属性为中心构建理论，采取一种以行为的相关属性为基础的方法定义行为，它也有助于开发行为分类法并对其进行理论化。因为行为的潜在属性不同，行为也各不相同；例如，有些行为会让人上瘾，有些行为是可以公开观察到的，有些行为则是代价高昂的。因此，成瘾性、隐私和成本是与行为理论相关的（众多属性中的）三个属性。我们描述了一个可以实施的以属性为中心的理论框架，其中包括生成行为属性、验证和测试这些属性，以及构建行为矩阵来为活动或干预提供依据。我们通过麦地那龙线虫病和心血管疾病的例子来阐明这一框架。这些方法有利于为干预的开发提供依据和概括不同的行为；然而，我们仍需要更多的研究将行为矩阵转化为实际政策。

## Резюме

Несмотря на важность поведения в укреплении здоровья и хорошего самочувствия, убедить людей принять и поддерживать здоровый образ жизни остается серьезной проблемой для здоровья населения. Значительный прогресс был достигнут в разработке и проверке теорий о личностных, социальных, экологических и структурных факторах поведения. Однако теоретические представления о самом поведении остаются труднодостижимыми, о чем свидетельствует отсутствие общепринятой таксономии поведения. Тщательно изучив природу поведения, практикующие врачи и исследователи могут определить наиболее эффективные способы содействия изменению поведения. В качестве подхода к определению поведения на основе его релевантных свойств предлагается теоретизирование с акцентом на атрибутах, которое затем может помочь в разработке таксономии поведения и построении теорий о нем. Модели поведения различаются по своим базовым свойствам; например, некоторые модели поведения вызывают зависимость, другие соблюдаются на людях, третьи дороги в осуществлении. Таким образом, способность вызывать зависимость, конфиденциальность и стоимость являются тремя (из многих) атрибутами, имеющими значение для построения теорий в отношении поведения. В статье описана схема практического применения теоретизирования, ориентированного на атрибуты, которая включает создание поведенческих атрибутов, проверку и тестирование этих атрибутов, а также построение поведенческой матрицы для информирования кампаний или вмешательств. Эта схема проиллюстрирована на примере дракункулеза и сердечно-сосудистых заболеваний. Среди преимуществ предлагаемого подхода — возможность информационной поддержки при разработке мер вмешательства и способность обобщать различные модели поведения, однако необходимо провести дополнительные исследования по преобразованию поведенческой матрицы в фактическую политику.

## Introduction

Health behaviours are critically important over a wide range of diseases and risk factors such as obesity, cancer, hypertension, unwanted pregnancy and human immunodeficiency virus (HIV) infection.^1^ Human actions underlie even seemingly intractable problems such as climate change and watershed protection.^2^ The current coronavirus disease 2019 pandemic has highlighted the importance of behavioural change in public health;^3^ minimizing infections to reduce transmission has only been possible as a result of the widespread adoption of face mask wearing, regular hand washing, physical distancing, testing and vaccination.^4^

Despite the substantial literature on behavioural drivers (i.e. the predictors of behaviour, which are not part of the behaviour itself),^5^ relatively little research has been conducted on the actual behaviours. Seminal behavioural theories predict behaviours based on the characteristics of individuals,^6^ social networks,^7^ social factors,^8^ environments^9^ and public policies.^10^ However, none of these perspectives provide guidance on how to promote behavioural change based on the characteristics of the focal behaviour itself. Because behaviours vary over multiple dimensions, efforts to change them must be adaptive to the underlying characteristics, that is, the attributes of the behaviour, distinct from behavioural drivers. 

Smoking cigarettes is an example of behaviour in which addictiveness is a key attribute.^11^ In many parts of the world, it is also an expensive behaviour and involves a restricted product. The three relevant attributes of smoking cigarettes are therefore addictiveness, cost and restrictiveness. However, addictiveness and cost are not attributes that define the wearing of face masks; in this case, availability and public visibility may be important attributes. Conversely, privacy considerations may be key for certain behaviours (e.g. contraception use), but not for others (e.g. physical activity). 

Behavioural attributes can also be categorized according to (i) temporality: some behaviours have immediate consequences (e.g. applying a bandage to an open wound) and others have more long-term effects (e.g. recycling household waste); (ii) the recipient of the benefits of the behaviour (e.g. satisfying cravings by eating high-sugar foods, or registering as an organ donor); and (iii) the required frequency of the behaviour (e.g. annual influenza vaccinations versus a single smallpox vaccination). 

Attribute-centred theorizing defines behaviour in terms of a unique configuration of its underlying behavioural attributes and the relative importance of these attributes.^12^ In this paper, we describe a framework for using attribute-centred theorizing in the development of public health interventions. We illustrate this framework using the case studies of Guinea-worm disease and cardiovascular diseases, and discuss the strengths and limitations of the technique. 

## Attribute-centred theorizing

We first introduced attribute-centred theorizing in 2011 (originally referred to as the attribute-centred approach).^12^ Several researchers have since used the approach to theorize about behaviours, in particular to model the conditions under which social norms drive people’s behavioural choices. For example, Manning^13^ identified four key behavioural attributes (social approval, social motivation, pleasantness and “interpersonal-ness”) to model the influence of social norms across 31 behaviours. Other researchers used attribute-centred theorizing to explain when and how behaviours are guided by social norms,^14^^–^^16^ to identify promising interventions across a variety of behaviours^17^^,^^18^ and to build behavioural theory.^19^ Using attribute-centred theorizing to guide public health interventions is a three-stage approach: (i) attribute generation, (ii) attribute testing and verification, and (iii) behavioural matrix construction.

### Attribute generation

The first stage is the generation of a list of relevant behavioural attributes using qualitative and mixed-method techniques. We recommend beginning by consulting key source documents – newspaper reports, journal articles, government briefs, social media feeds and other channels – for descriptions of the behaviours in question, noting the manifest attributes. In the case of vaccination against severe acute respiratory syndrome coronavirus 2, such preliminary searches could yield the information that vaccines require government approval and are in short supply, and that complete vaccination requires two injections; the key attributes are therefore scarcity, the requirement of approval and repetitiveness.

Attributes can also be generated through thought-listing exercises, or asking participants key questions that are designed to elicit important properties of the behaviour. For example, participants might be asked to (i) list thoughts that come to mind when you hear the word vaccination; (ii) say what happened when you received your last vaccine; or (iii) describe what you remember reading about vaccines recently. Responses to these prompts are used to delineate the key attributes pertaining to vaccination.

### Attribute testing and verification

Attributes generated in the first step then undergo testing and verification. In surveys, participants can rate the importance of each attribute identified in the first step for different behaviours through scaled responses. For example, if the attribute is addictiveness, participants can be asked how important it is for smoking, exercising, wearing a mask, vaccination, screening and any number of other behaviours of interest. Similarly, participants can be asked to rate the attribute’s relevance, usefulness or salience for each focal behaviour. This method can be done across a variety of behaviours and attributes,^20^ or for a single behaviour and attribute believed to be very important (e.g. hand washing visibility).^21^ The results are averaged to determine how each attribute is associated with each focal behaviour, and analytical methods used to assess how various attributes cluster together and how they are related, as groups, to various behaviours.

Although this survey method can lead to meaningful quantification of the relationships between attributes and behaviours, its primary drawback is that it is often obtrusive and prone to error for sensitive or stigmatizing behaviours. To increase accuracy, this method can be supplemented with other unobtrusive methods such as the approach-avoidance task (Box 1). The approach-avoidance task proposes that it is cognitively easier to process attributes consonant with the underlying behaviour than attributes discordant with the behaviour.^24^

Box 1 **The approach-avoidance task and its relevance to attribute-centred theorizing**

Human brains process certain kinds of information more easily than others, and the speed with which information is processed is often taken as an indication of this ease (or difficulty). When information is easy to process, cognitive burden is low and people’s reaction time (often measured in milliseconds), or the time needed to process that information, is shorter; conversely, when tasks are more complex, reaction time is longer because the cognitive burden is greater. Cognitive burden, in turn, is greater when the stimulus is characterized by conflict or if it inspires negative emotions such as fear.^22^

The approach-avoidance task is used to assess reaction times in laboratory-based experiments. Typically, people are asked to pull a lever (or press a particular button) as soon as they see something specific as instructed. For example, people might be asked to pull the lever as soon as they see the colour green or the letters g-r-e-e-n on their screen. The reaction time is typically longer when there is conflict (e.g. if the word “green” is written in red ink) than when there is no conflict (e.g. the word “green” is written in green ink). This disagreement is known as stimulus conflict, which typically garners a longer response time.^23^

This example illustrates the idea that concordant information (green colour to spell the word “green”) garners shorter reaction time than discordant information (red colour to spell “green”). Extending this notion to attribute-centred theorizing leads to the proposition that when people process an attribute that is concordant with the behaviour in question (e.g. need for privacy as an attribute of HIV testing), their reaction time, as measured through an approach-avoidance task, would be shorter than when they process a discordant attribute (e.g. testing for HIV in a public setting).

### Behavioural matrix construction

The next step is the construction of the behavioural matrix, which shows how each attribute (row) is associated with a given behaviour (column); the contents of each cell describe the extent to which a particular attribute is important for the given behaviour (Table 1). This matrix can be further augmented by calculating cell entries, which are specific weights across behaviours for each attribute. For example, instead of merely noting that the strength of relationship between the attribute of repetitiveness and behaviour of wearing a mask is high, conjoint analysis techniques^25^ can be used to quantify relative weights. Construction of a precise and accurate behavioural matrix is achieved iteratively across many studies in diverse contexts.

**Table 1:** The behavioural matrix in attribute-centred theorizing: strength of relationship between attributes and behaviours for control of coronavirus disease transmission

Attribute	Wearing masks	Washing hands	Physical distancing	Testing	Vaccination
Repetitiveness	High	High	High	Medium	Low
Cost	Low	Low	Low	Low	Medium
Public visibility	High	Low/high^a^	High	Medium	Medium

^a^ The matrix could also provide empirically derived weights describing the relationship between each attribute and the focal behaviour. Values may also differ by context. For example, visibility of washing hands may be ranked high in public facilities, but not always in private.

The overall purpose of a behavioural matrix is to guide the development of intervention, allowing public health campaigns to receive empirically based advice on which attributes should be emphasized to change particular behaviours and behavioural clusters. For example, regular hand washing and social distancing are both behaviours that are visible to others, low in cost and repetitive; a campaign emphasizing the social norms supportive of the behaviours, easy access and the ritualistic nature of the behaviours might therefore be a useful approach when used in combination with structural and environmental changes to facilitate these behaviours.^21^

## Illustrations

We illustrate attribute-centred theorizing using the examples of Guinea-worm disease and cardiovascular diseases. These examples were chosen because of their behavioural foci and range of behavioural attributes.

Guinea-worm disease is caused by the parasitic worm *Dracunculus medinensis*. The disease is endemic in Guinea and in several countries across the world; however, this disease can be eliminated by changing human behaviour, and it is on the verge of being eradicated without the use of drugs.^26^^–^^29^ Indeed, it has been noted that the demise of Guinea-worm disease “will be proof that people can be persuaded to change their behaviour through innovative health education.”^30^

The World Health Organization defines cardiovascular diseases as “a group of disorders of the heart and blood vessels,” for which the leading behavioural risk factors are unhealthy diet, physical inactivity, tobacco use and harmful levels of alcohol consumption.^31^ Although pharmacological interventions can be a part of the treatment, both prevention and risk reduction involve significant individual lifestyle changes.^32^

Because Guinea-worm disease is a neglected tropical disease, there may be relatively few popular press articles or published studies on related behaviours; it may therefore be necessary to research other similar behaviours for different diseases for clues to the attributes. In contrast, cardiovascular diseases are more common and discussions of underlying behaviours, and hence the salient attributes, may be more readily discernible (Table 2). 

**Table 2:** Illustration of attribute-centred theorizing for the development of behavioural change interventions to reduce prevalence of Guinea-worm disease and cardiovascular diseases

Attribute-centred theorizing stage	Guinea-worm disease	Cardiovascular diseases
Attribute generation	Required risk reduction behaviours: treating water sources, appropriately disposing of aquatic animal waste, tethering or containment of animals, and identifying and treating infections in humans and animals^28^	Required risk reduction behaviours: regular fruit and vegetable consumption, regular physical activity, smoking cessation and reduction of salt intake^32^
	Associated attributes: requirement of technical knowledge, collective benefit, requirement of resources and complexity	Associated attributes: visibility, individual benefit, addictiveness
Attribute testing and verification	Surveys of community members	Social media search
Behavioural matrix construction	e.g. Treatment of water sources may be high for requirement of technical knowledge, high for collective benefit and high for requirement of resources	e.g. Daily consumption of fruits and vegetables may be low for visibility to others, high for individual benefit and low for addictiveness

Observations of people enacting the behaviours and discussions with community members about the behaviours themselves are other means of generating relevant attributes. The goal of these activities is to understand how the behaviours are enacted and understood by people in the community, which helps to identify relevant attributes. Visibility could be an important attribute in both examples (tethering animals for Guinea-worm disease or conducting physical exercise for cardiovascular disease), whereas the requirement for technical knowledge may be greater for treating water than for eating fruit and vegetables. Similarly, the extent to which the behaviour confers benefits collectively (e.g. improving the health of the community by providing access to treated water) or individually (e.g. improving health by not smoking), the frequency with which the behaviour needs to be conducted (the regular and continuous containment and care of animals or engagement in physical activity) and whether the behaviour is associated with addiction (not relevant for tethering animals but relevant for smoking) are other attributes for consideration (Table 2).

Once attributes have been identified, their relevance for the behaviour at hand needs to be tested. In this step, the goal may be to determine the extent to which people perceive the behaviour in question as comprising the attributes or the extent to which the attributes are salient or relevant. Surveys of community members in areas in which Guinea-worm disease is endemic could be undertaken. Likewise, searching social media for content from groups of people with heart disease (e.g. the social networking website PatientsLikeMe®)^33^ can provide evidence for the salience of attributes. One of the qualitative methods available for testing and verification includes the free-listing exercise, which can identify barriers to particular behaviours.^20^ “Tell me what comes to mind when you think about tethering your animals before you head out to the fields,” for example, can serve as an open-ended question designed to determine whether the attributes identified by the researchers or programme planners also match those emerging in people’s conversations. Another qualitative method is a pile-sorting exercise,^34^ in which participants categorize different attributes in terms of their importance or relevance.

Finally, the behavioural matrix is constructed to describe the strength of the relationship between the attribute and the behaviour. Table 2 lists the strengths of the relationships between: (i) the attributes of requirement of technical knowledge, collective benefit and requirement of resources, and the behavioural example of disposal of aquatic animal waste (Guinea-worm disease); and (ii) the attributes of visibility, individual benefit and addictiveness, and the behavioural example of daily consumption of fruit and vegetables (cardiovascular diseases). 

Analysis of behavioural attributes using this technique can identify the extent to which potential interventions to influence behaviours related to the prevalence of Guinea-worm disease or cardiovascular diseases are likely to be effective. For example, if an intervention under consideration includes a communication campaign to describe the social norms associated with a behaviour, an action that is visible to others in the community, undertaken collectively and simple to implement is more likely to be adopted.^35^

## Discussion

The attribute-centred theorizing approach has several benefits for public health interventions. First, aligning relevant behavioural attributes with theoretical assumptions provides guidance on which theory to adopt in the promotion of behavioural change. For example, if a particular theory is based on an assumption of individual desire in enacting change,^36^ then its use for behaviours defined by addictiveness would be unwise. Conversely, theories about social norms (typically based on people’s observations and perceptions about others’ behaviours)^35^^,^^37^ might be more relevant for changing behaviours defined by high public visibility.^38^

A second benefit of the approach is that it allows for the inclusion of behavioural clusters, beyond individual behaviours, in programme planning and implementation. When multiple behaviours confer benefits in terms of risk or harm reduction, identifying those with similar attributes can make interventions more efficient.^39^

A third benefit relates to the generalizability of the approach across behaviours. Because prior behavioural change theories have not focused on the underlying attributes, generalizing beyond the settings and populations from which the findings of a study are derived is difficult. This situation has led to higher levels of confidence in theories about people and environments, but lower confidence in theories about behaviours themselves. Attribute-centred theorizing provides a framework that allows for the accumulation of evidence about how attributes uniquely define behaviours. For example, as shown in Table 1, washing hands is a behaviour defined by the attributes of repetitiveness (high strength of relationship), cost (low strength) and public visibility (low or high strength, depending on context). These same attributes also define another behaviour (wearing masks), but their strengths differ (high, low and high, respectively). By accumulating evidence of the differential weights of attributes in defining behaviours empirically across multiple studies in multiple contexts, theories about behaviours themselves can be developed.

Our approach of attribute-centred theorizing has two main limitations. The first is the difficulty in drawing the distinction between attributes and predictors of behaviours. For example, perceived benefits are often drivers of behaviours; people are more likely to act if they perceive benefits from doing so. However, this variable can be confused with behavioural attributes. The second limitation is the difficulty in translating the behavioural matrix into interventions. Nevertheless, we anticipate that this is a function of the novelty of the approach, and it is hoped that future researchers and practitioners can contribute to this effort in a significant way.

To conclude, attribute-centred theorizing is an empirically based practical tool for behavioural change. Its adoption can focus interventions, reduce redundancy across health domains, and better leverage existing public health research and practice. It can be applied formally when the time, resources and capacity exist to do so, or informally; in either case, it provides a structured method of carefully examining the nature of behaviour when behavioural change interventions are being considered.
